# Comparative Evaluation of the Effectiveness of Novel Hyaluronic Acid-Polynucleotide Complex Dermal Filler

**DOI:** 10.1038/s41598-020-61952-w

**Published:** 2020-03-20

**Authors:** Jong Hwan Kim, Tae-Rin Kwon, Sung Eun Lee, Yoo Na Jang, Hye Sung Han, Seog Kyun Mun, Beom Joon Kim

**Affiliations:** 1 Department of Dermatology, Chung-Ang University College of Medicine, Seoul, Korea; 2 Department of Medicine, Graduate School, Chung-Ang University, Seoul, Korea; 3 Department of Otorhinolaryngology-Head and Neck Surgery, Chung-Ang University College of Medicine, Seoul, Korea

**Keywords:** Outcomes research, Preclinical research

## Abstract

HA (Hyaluronic acid) filler, the most commonly used dermal filler, causes several side effects. HA-PN (Hyaluronic acid-Polynucleotide), a new composite filler, has excellent biocompatibility and induces tissue regeneration. In this study, we compare the efficacies and safety profiles of these fillers. The characteristics of HA and HA–PN fillers were compared using scanning electron microscopy and rheometry. No morphological difference was noted between the fillers. However, the latter had higher viscosity and elasticity values. The HA-PN filler induced higher cell migration than the HA filler in a wound healing assay. It was also found to stimulate better collagen synthesis in human and mouse fibroblasts. The HA and HA–PN fillers were injected into SKH1 hairless mice to determine changes in their volume for up to 24 weeks. Increased cell migration and collagen synthesis were observed in mice injected with the HA–PN complex filler. Although the safety and durability of the HA and HA–PN fillers were similar, the latter induced a lower transient receptor potential vanilloid 4 expression and caused less stimulation upon injection. In conclusion, HA–PN complex fillers can stimulate fibroblast growth and facilitate volume growth and skin regeneration.

## Introduction

Dermal fillers can be used in simple and short procedures in surgery and rapid facial rejuvenation. There is growing interest in the use of fillers for tissue enlargement and improvement of skin aesthetic beauty^1–3^.

Synthetic facial fillers are composed of a biosynthetic polymer in combination with different injectable carriers, including hydrogels, beads, and liquids^4^. The most popular type of filler contains hyaluronic acid (HA). However, the use of HA fillers carries an inherent risk of hypersensitivity reactions because these fillers contain hyaluronan-related proteins and 1,4-butanediol diglycidyl ether (BDDE)^5,6^. In addition, small fragments of HA can cause inflammation and elicit adverse effects that include erythema, slight oedema, hematoma, itching, and pain^7–9^. A recent study showed that the biocompatibility of HA-based materials decreases with an increase in the number of modifications to this polysaccharide^5,6,10^.

Various dermal fillers have been developed to overcome the adverse effects accompanying the use of HA fillers^6,11–14^. Recently, a new filler product was synthesised using a purified polynucleotide (PN) extracted from salmon and other fish germ cells. This product is currently in use in Europe^15^. While existing filler products work by simply filling spaces in the skin^16^, PN-containing products also induce the regeneration of damaged tissues to result in a more natural tissue regeneration^15^. In addition, it has been reported that nucleotides promote the growth of human corneal fibroblasts and increase their remnants in ultraviolet B-damaged skin fibroblasts^17^. *In vitro* studies have demonstrated the therapeutic efficacy of polynucleotides in patients who received treatment for skin ectopia and have shown that polynucleotides promote rapid corneal epithelialisation after photorefractive keratectomy^18,19^.

The use of a PN filler offers the advantages of skin elasticity, collagen synthesis, and regeneration by stimulation of fibre elasticity. However, the volumising effect and durability of PN dermal fillers are not as good as those of existing dermal fillers^15,20,21^. In this study, we used an HA–PN (Hyaluronic acid-Polynucleotide) complex filler, which has the advantages of both tissue regeneration and durability. This filler overcomes the disadvantages of its individual components. This study also aimed to demonstrate the commercial feasibility of the HA–PN complex filler by comparing the efficacies and safety profiles of HA and HA–PN complex fillers *in vitro* and *in vivo*.

## Results

### Scanning electron microscopy (SEM) analysis of HA and HA–PN complex fillers

**Figure 1:**
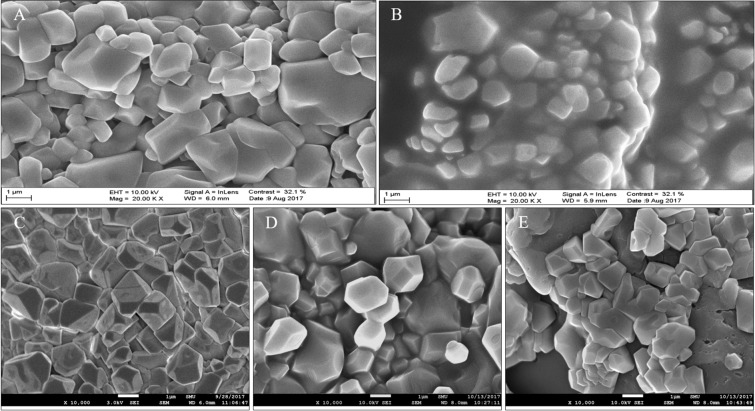
SEM images of HA and HA–PN fillers. (**A**) HA filler, Juvéderm VOLUMA. (**B**) HA filler, Juvéderm VOLBELLA. (**C**) HA–PN (0.1%) complex filler. (**D**) HA–PN (0.5%) complex filler. (**E**) HA–PN (1%) complex filler.

The morphologies of HA and HA-PN complex fillers were compared using SEM. Based on the SEM images, both fillers are made up of irregular and small polygonal particles (Fig. 1).

### Comparison of rheological properties of HA and HA–PN fillers

The G′ mean values of elasticity, G″ mean values of viscosity, G* mean values of viscoelasticity, and the mean values of tan δ, indicating the ratio of viscosity to elasticity, were measured (Supplementary Fig. 1, Table 1). All values were higher for the HA–PN complex fillers than for the HA fillers. These results confirmed that the addition of PN to HA increases the filler’s elasticity, viscosity, viscoelasticity, and modulus of elasticity.

**Table 1:** Storage modulus (G′), loss modulus (G′′), complex viscosity (η*), and phase angle (δ) measured at a frequency of 1 Hz at 25 °C.

	G′ (Pa)	G″ (Pa)	G* (Pa)	δ (°)
Juvederm VOLUMA^®^	340.97 ± 27.66	37.63 ± 5.25	343.12 ± 27.28	6.36 ± 1.19
Juvederm VOLBELLA^®^	196.10 ± 24.82	32.91 ± 3.15	198.93 ± 24.36	9.69 ± 1.73
HA-PN 0.1%	1107.15 ± 281.26	448.64 ± 42.64	1197.48 ± 271.95	22.84 ± 3.80
HA-PN 0.5%	1165.65 ± 237.13	323.56 ± 26.58	1210.48 ± 234.30	15.88 ± 2.06
HA-PN 1%	509.66 ± 87.55	132.56 ± 21.92	526.63 ± 90.17	14.59 ± 0.42

### Comparison of cell cytotoxicity and proliferation induced by HA, PN, and HA–PN treatments

**Figure 2:**
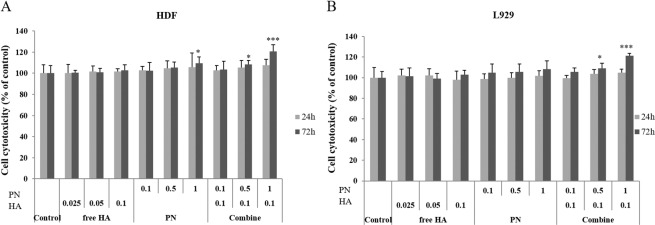
MTT assay to determine the viability of (**A**) HDF cells and (**B**) L929 cells after treatment with increasing concentrations of HA, PN, and HA–PN fillers for 24 h and 72 h. Statistical significance was determined by the Student’s *t*-test. *P < 0.05, **P < 0.005, ***P < 0.0005.

To assess the cytotoxic effects of HA, PN, and HA–PN treatments on human dermal fibroblast (HDF) and mouse fibroblast (L929) cells, comparative cytotoxicity tests using the MTT (3-(4, 5-dimethylathiazolyl-2)-2, 5-diphenyltetrazolium bromide) assay were performed. The cells were incubated with the fillers for 24 and 72 h before cytotoxicity was assessed (Fig. 2). HA, PN, and HA–PN did not display dose- or time-dependent cytotoxic effects. On the contrary, the proliferation of HDF cells was evident following treatment with only 1% PN, a combination of 0.1% HA and 0.5% PN, and a combination of 0.1% HA and 1% PN. Additionally, the proliferation of L929 cells increased significantly after treatment with a combination of 0.1% HA and 0.5% PN and a combination of 0.1% HA and 1% PN. In particular, co-treatment with 0.1% HA and 1% PN increased the proliferation of HDF and L929 cells by approximately 20%.

### Comparison of cell migration following HA, PN, and HA–PN treatment

**Figure 3:**
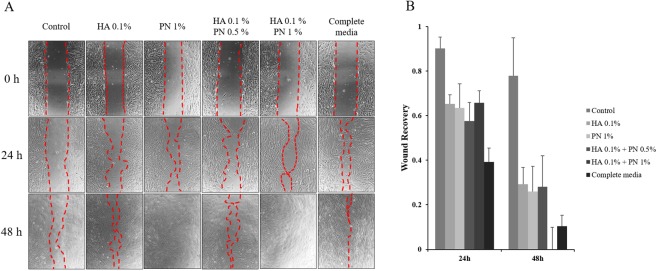
Effect of PN on the migration of human fibroblast cells. Wound Healing Analysis Images (Zoomth increasing concentrations of Ibidi chambers and cultured to near confluence. A free ‘scratch’ or wound was created on the bound monolayer of cultured cells. The cells were incubated in medium containing 0.1% HA, 0.5% PN, or 1% PN for 0, 24 and 72 h at 37 °C in a humidified incubator with a 5% CO2 atmosphere. Medium Supplemented with 10% FBS was used in the complete medium group. The wound areas were quantified and expressed as a percentage of the initial wound area.

To further investigate the effects of HA, PN, and HA–PN on the proliferation of HDFs, a wound healing assay was performed after HA, PN, and HA–PN treatments. After incubation for 48 h, cell migration was confirmed (Fig. 3A). In particular, co-treatment with 0.1% HA and 0.5% PN showed the highest cell growth potential (Fig. 3B). These results suggest that PN is effective for cell proliferation.

### Comparison of collagen synthesis by HA, PN, and HA–PN treatments

**Figure 4:**
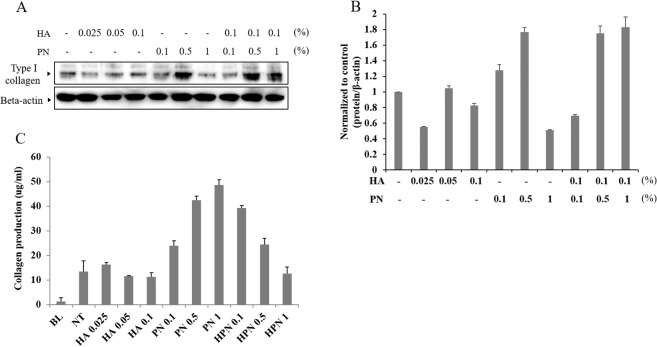
Quantification of the amount of type I collagen expressed in HDF cells. (**A**) Western blot image. (**B**) Quantification of the type I collagen expression in HDF cells. (**C**) Quantification of soluble collagen in the cell culture supernatant using Sircol collagen assay.

To determine the correlation between proliferative capacity and collagen expression, the expression of type I collagen at the protein level was analysed by western blotting using HDF cells treated with HA, PN, or HA–PN. Collagen expression was higher in the 0.5% PN group and the 0.1% HA and 0.5% or 1% PN co-treatment groups when compared to that in untreated HDF cells (Fig. 4A,B). Soluble collagen in cell culture supernatants was quantified using the Sircol collagen assay. A dose-dependent increase in collagen synthesis was observed in the PN group and the HA-PN co-treatment group, but not in the HA group (Fig. 4C). Therefore, PN treatment induced an increase in collagen synthesis in the cells.

### Comparison of durability between HA and HA–PN complex fillers *in vivo*

**Figure 5:**
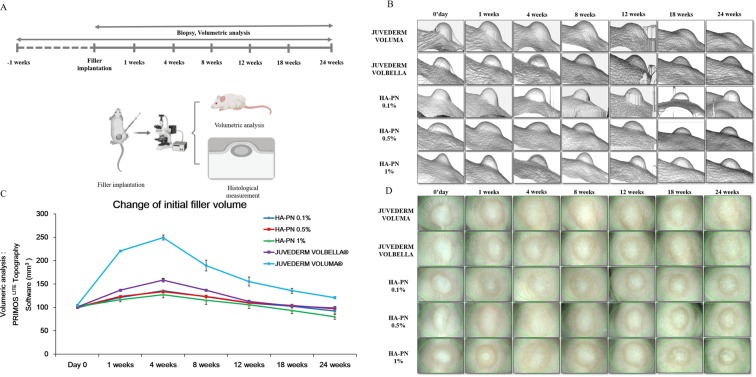
Evaluation of durability of HA and HA–PN complex fillers in vivo. (**A**) Study design scheme(Created with BioRender). (**B**) Image depicting volume change after the filler injection was taken using PRIMOS. (**C**) Graph showing volume change after filler injection. (**D**) A folliscope image depicting volume change after filler injection.

To confirm the durabilities of the HA and HA–PN fillers, 100 μL of each filler was injected into the dorsal region of mice, and changes in the volumes of the fillers were observed. Both HA and HA–PN fillers displayed an increase in volume for up to 4 weeks. The largest volume was observed for Juvéderm VOLUMA (250 mm^3^), followed by those for Juvéderm VOLBELLA (158 mm^3^), HA–PN 0.1%, 0.5% (134 mm^3^), and HA–PN 1% (127 mm^3^). These results suggested that the higher the concentration of HA, the larger is the volume growth rate. In addition, although the HA concentrations (1.5%) for Juvéderm VOLBELLA and the HA–PN complex filler were the same, the volume growth rate decreased in a dose-dependent manner for PN. Both injection fillers decreased in volume over time from 4 weeks after filler injection to 24 weeks of evaluation. The volume changes were as follows: Juvéderm VOLUMA, 120 mm^3^; Juvéderm VOLBELLA and HA–PN 0.5%, 98 mm^3^; HA–PN 0.1%, 92 mm^3^; HA–PN 1%, 97 mm^3^ (Fig. 5).

### Comparison of histological analysis results between HA and HA–PN complex fillers

**Figure 6:**
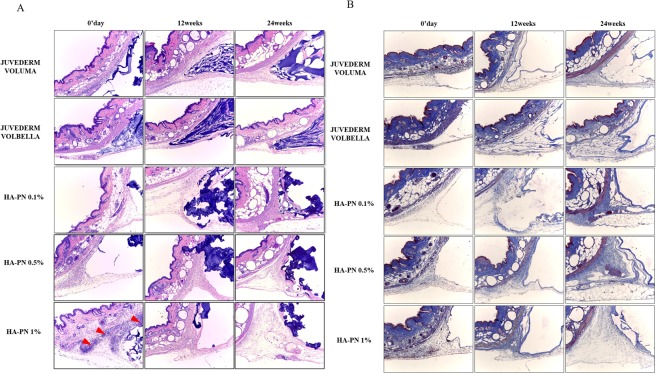
Histological evaluation of HA and HA–PN fillers. (**A**) Identification of inflammation and foreign body reaction by hematoxylin and eosin staining at Day 0, Week 12 and Week 24. (**B**) Identification of collagen production using Masson’s trichrome staining at Day 0, Week 12 and Week 24.

To confirm the degree of inflammation and reaction with foreign bodies *in vivo*, skin tissue at the filler injection site was biopsied at 0 h, 12 weeks, and 24 weeks. Haematoxylin and eosin-stained tissue slides were observed at 100× magnification using an optical microscope, and the main histological features of each slide were determined. Histopathological evaluation showed that there was no reaction to foreign bodies upon the injection of both fillers (Fig. 6). However, with the 1% HA–PN complex filler, the presence of inflammatory cells was detected immediately following filler injection. No inflammatory cells were observed at week 12. Collagen synthesis was confirmed after staining with Masson’s trichrome. Thus, compared to the HA filler, the complex filler containing PN was associated with greater collagen synthesis.

### Comparison of transient receptor potential vanilloid 4 (TRPV4) expression between HA and HA–PN complex fillers by immunofluorescence analysis

**Figure 7:**
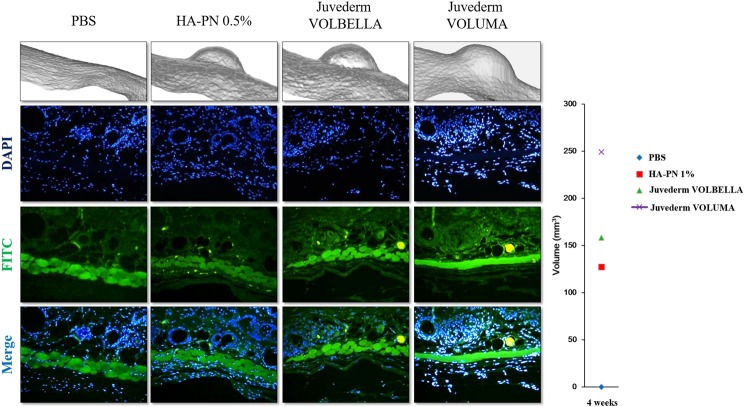
Comparison of TRPV4 expression after treatment with HA and HA–PN fillers using immunofluorescence staining of mouse tissue. The extent of stimulation in tissues by TRPV4 at Week 4 was determined using immunofluorescence staining. The increases in volume as a result of filler treatments were also compared.

TRPV4 tissue staining was performed to determine the degree of stimulation in the tissue owing to filler injection. TRPV4 mediates pain-related behaviour caused by mild hyperactivity in the presence of inflammatory mediators. Immunofluorescence staining was confirmed at 4 weeks, and the maximum volume was measured after each filler injection. TRPV4 expression increased in the muscle layer after treatment with the HA fillers Juvéderm VOLUMA (2.0%) and Juvéderm VOLBELLA (1.5%). On the contrary, the TRPV4 expression level after HA–PN 1% complex filler treatment was equivalent to that for treatment with the negative control, PBS (Fig. 7).

## Discussion

The terms “biological stimulation” and “biological regeneration” have been used to describe the functions of many aesthetic medical devices^22^. Regeneration is the process of restoration and growth that makes the genome, cell, organism, and ecosystem resilient to events that cause natural fluctuations, disturbances, or damages^23,24^. Several clinical studies have described the therapeutic use of polynucleotides in skin regeneration and wound healing^25–28^. The HA–PN complex filler, composed of HA and PN polymer, is a new formulation for skin regeneration and tissue restoration. We confirmed the improved functions of the HA–PN complex filler through comparisons with the Juvéderm VOLUMA and Juvéderm VOLBELLA high-volume HA fillers. Moreover, we further analysed and compared all these fillers in terms of their morphological and rheological properties, and their effects in cells and animal models.

Polynucleotides are widely distributed in the human body and exist physiologically in the extracellular environment^29^. They readily bind to water molecules and act as free radical scavengers^30,31^. The nutritional effects of PN have been shown in multiple *in vitro* studies using human fibroblasts in primary cultures. The ability of PN to stimulate the secretion of collagen proteins and other proteins in the extracellular matrix has also been demonstrated^32–34^.

The HA–PN complex filler is morphologically similar to the HA filler. However, we confirmed that its rheological properties, in terms of G *, G′, G′′, and tan δ, are different from those of the HA filler. It was confirmed that the hardness, elasticity, viscosity, and viscoelasticity of the cross-linked gel are dependent on the PN content of the HA–PN complex filler. In addition, no toxicity was associated with PN or HA-PN treatments in human and mouse fibroblast cells. Moreover, in human fibroblast cells, the content of type I collagen increased with the increasing PN concentration. However, excess PN inhibited the synthesis of type 1 collagen.

In animal studies, the HA–PN complex filler showed the greatest increase in collagen synthesis and maintained a natural volume, with no significant increases in the initial filler injection volume. In addition, previous studies have identified six TRPV proteins as receptors for stimulation of neurons^35,36^. Among these TRPV proteins, TRPV4 is a ubiquitously expressed plasma membrane-based calcium-permeable cation channel that is sensitised and activated by chemical and physical stimulation^37–40^. When skin is stimulated, the expression of matrix metalloproteinases (MMPs) and inflammatory cytokines can increase^41,42^. The increased levels of MMPs result in the degradation of collagen and elastic fibres in the skin and promote skin aging^43,44^. In this study, HA filler containing PN induced a lower expression of the TRPV4 protein, a neuronal stimulatory receptor, compared to HA filler. When PN was included, it induced a reduced stimulation and lower MMP and inflammatory cytokine expression. This finding indicated that HA fillers containing PN are more effective in inhibiting skin aging compared to HA fillers. Numerous fillers have been developed and used to achieve simple volume effects. However, HA–PN complex fillers can stimulate fibroblast growth for skin rejuvenation and have the added benefits of volume enhancement and skin regeneration. In addition, the trend is currently shifting from the provision of components (such as collagen, HA, and glycoproteins) into the skin to the stimulation of cellular components (such as fibroblasts) to enhance regeneration. To the best of our knowledge, this is the first study demonstrating the durability, efficacy, and safety of HA–PN complex fillers. We believe that our results can potentially be the next-generation paradigm in the filler market.

## Methods

### Materials

The HA-PN complex filler was supplied by NutraPharmTech (Seoul, Korea). It was prepared by mixing in the PN solution after sterilising the HA-based filler, which is generally cross-linked by BDDE. HA fillers made by Allergan were purchased from Juvéderm VOLUMA and Juvéderm VOLBELLA (Pringy, France). Type I collagen antibody (ab34710) and transient receptor potential vanilloid (TRPV4; ab39260) proteins were obtained from Abcam (Cambridge, Mass.).

### Scanning electron microscopy

The morphology of each filler was evaluated using SEM. Each filler was diluted with water for injection (WFI), filtered through a 0.22 μm membrane filter mounted in a stainless-steel filter housing, and then placed in an oven until the filter contents were completely dry. Filler samples were mounted on stainless steel SEM pedestals pre-labelled with double-sided adhesive mounting disks. Samples were prepared by coating with gold-palladium powder and conventionally imaged using a LEO SUPRA 55 microscope (Carl Zeiss, Jena, Germany).

### Rheological measurements

The storage modulus (G′) and viscous modulus (G″) were measured using a rheometer (Kinexus Pro, Malvern, UK). G′ characterises the stiffness of the gel – a stiff material has a higher G′ than a soft material. All measurements were carried out using a 20-mm steel plate oscillating at a frequency between 0.1 and 10 Hz. The values presented were compared to the average of the values obtained at frequencies of 1 to 10 Hz. Measurement conditions were as follows: oscillation mode; a shear strain of 1.5%; a frequency of 0.05 to 25 Hz; an interval of 0.5 mm; and a temperature of 25 °C.

### Cell maintenance

HDF cells (CCD-25SK; American Type Culture Collection, Manassas, VA, USA) were grown in Dulbecco’s modified Eagle’s medium (DMEM) Supplemented with 10% foetal bovine serum (FBS), 2 mM l-glutamine, 100 U/mL penicillin, and 100 mg/mL streptomycin, at 37 °C in a humidified incubator with a 5% CO_2_ atmosphere^45^. In addition, an established mouse fibroblast cell line (L929, catalogue code CCL-1; American Type Culture Collection) was cultured in minimal essential medium Supplemented with 5% foetal calf serum, 100 U/mL penicillin, 100 μL/mL streptomycin, and 2 mmol/L l-glutamine at 37 °C in a humidified incubator with a 5% CO_2_ atmosphere^46^.

### Cell viability

The MTT assay was performed to measure the cell viability of the HDFs and L929 mouse fibroblasts. HA (0.025% to 0.1%) and PN (0.1% to 1%) were added after 5 h of cell culture. MTT solution (5 mg/mL) was added and incubated at 37 °C for 4 h. After incubation, the supernatant was removed, and the formazan formed by MTT reduction was dissolved in dimethylsulfoxide^47^. The absorbance of formazan was measured at 570 nm using a SoftMax Pro5 ELISA microplate reader (Molecular Devices, Sunnyvale, CA, USA).

### Wound healing assay for cell mobility

The wound healing assay was performed based on previous studies by measuring cell migration. The cell migration is defined as the time taken to close an open wound after a linear ‘scratch’ or wound is created across a monolayer of cultured cells^48,49^.

Ibidi culture-inserts (μ-Dish 35 mm, high culture-inserts; Thistle Scientific Ltd., Glasgow, UK) were used for the assay. The sample was diluted in DMEM and was used to treat the cells, which were then cultured for 24 to 72 h. DMEM Supplemented with 10% FBS was used as a positive control. After 48 h, the movement of cells was photographed using an Eclipse TS100 inverted microscope (Nikon Instruments Inc., Tokyo, Japan). Images were normalised using the XnConvert software and wound sites were quantified using the Image J software (ImageJ; version 1.51 i; U.S. National Institutes of Health). The final wound area was quantified and expressed as a percentage of the initial wound area. This represented the degree of wound healing at that point in time.

### Western blotting

HDF cells were cultured in DMEM Supplemented with FBS for 24 h. The treated cells were exposed to different concentrations of HA and PN in the absence or presence of FBS. Every hour during the culture, the treated and control cells were collected and a defined amount of total protein, as determined using the Bradford protein assay (Bio-Rad Laboratories, Hercules, CA, USA), was resolved using 10% SDS-PAGE. The resolved proteins were transferred to a polyvinylidene fluoride membrane. After the application of primary antibodies and conjugated secondary antibodies, each membrane was washed thrice for 10 min with Tris-buffered saline containing 0.1% Tween 20. The degree of protein expression was determined using ECL detection reagents (Thermo Fisher Scientific, Pierce Biotechnology, Waltham, MA, USA).

### Collagen measurement

The total soluble collagen from cell culture supernatants was quantified using the Sircol collagen assay, according to the manufacturer’s instructions (Biocolor, Belfast, UK)^50^. The absorbance was then measured at 555 nm, as it is directly proportional to the amount of collagen present in the cell culture medium. The soluble collagen was then hydrolysed in HCl, and the hydroxyproline levels were measured by a colourimetric method using an assay kit (QuickZyme Biosciences, Burlington, NC, USA), according to the manufacturer’s instructions^51,52^. The total collagen content was calculated from the hydroxyproline content of collagen standards^50^.

### Study design

All animal procedures were performed in accordance with the Guidelines for the Care and Use of Laboratory Animals of Chung-Ang University and approved by the Animal Ethics Committee of Chung-Ang University IACUC (Approval No. 201700022). Six-week-old SKH1 hairless mice were bred under temperature cycles of 24 ± 2 °C, 50 ± 10% humidity, and 12 h day/night cycles in the laboratory. The filler (100 µL) was injected into the dorsal skin of the posterior limbs of hairless female mice (n = 5 mice per group).

### Volumetric analysis

The volume of filler injected into each dorsal skin was measured in the anesthetised mice. After filler injection, the increase in volume was determined using a PRIMOS device at 0, 1, 4, 8, 12, 18, and 24 weeks. The volume ratios were calculated by comparing the volume and height measurements at each time point to the measurements on Day 0. The change in volume was quantitatively determined through three-dimensional (3D) measurements of the skin region at the different time points before and after treatment and computer-assisted comparison of the measured profile^53^.

### Histological analyses

Paraffin-embedded tissue sections were deparaffinised and stained according to previously published protocols^54^. Skin samples were fixed in 10% phosphate-buffered formaldehyde, embedded in paraffin, and processed for histological analysis. Sections, 5 μm in thickness, were sliced and mounted on slides. The sections were stained with haematoxylin and eosin (to confirm inflammation and foreign body reaction) and Masson’s trichrome (to confirm collagen biosynthesis), according to standard procedures.

### Immunofluorescence analysis

Paraffin-embedded tissue sections were deparaffinised and stained according to previously published protocols^55^. Anti-Transient Receptor Potential Cation Channel Subfamily V Member 4 (TRPV4) antibody (ab39260, 1:200; Abcam) was used as the primary antibody. After treatment with a blocking buffer containing 2.5% bovine serum albumin (BSA, Sigma-Aldrich, St. Louis, MO, USA) and 2.5% horse serum, the cells were incubated at 25 °C for 4 h. The tissues were washed with phosphate-buffered saline containing 0.1% Triton X-100 (PBST). A fluorescein isothiocyanate-goat anti-rabbit secondary antibody, which exhibits green fluorescence, was added to the blocking buffer containing 2.5% BSA and left in a dark room for 2 h.

### Statistical analyses

Statistical significance was computed using SPSS version 21 software (SPSS Inc., Chicago, IL, USA). One-way ANOVA and two-tailed unpaired *t*-tests were performed. *p ≤ 0.05, **p ≤ 0.005, ***p ≤ 0.0005.

## Supplementary information

Supplementary Figure 1.

## Data Availability

All data generated or analysed during this study are included in this published article and its Supplementary Information Files. Extra data are available from the corresponding author upon request.
