# Supplementary material for: Comparative Evaluation of the Effectiveness of Novel Hyaluronic Acid-Polynucleotide Complex Dermal Filler
Source: Sci Rep. 2020 Mar 20;10:5127. doi: 10.1038/s41598-020-61952-w (PMC7083941; doi:10.1038/s41598-020-61952-w)
Supplement: Supplementary file 1 — Supplementary Figure 1. [file 41598_2020_61952_MOESM1_ESM.pdf]

Comparative Evaluation of the Effectiveness of Novel Hyaluronic Acid-Polynucleotide Complex Dermal Filler

Jong Hwan Kim[1], Tae-Rin Kwon[1], Sung Eun Lee[1,2], Yoo Na Jang[1,2], Hye Sung Han[1,2], Seog Kyun Mun[3], Beom Joon Kim[1,2,*]

[1]Department of Dermatology, Chung-Ang University College of Medicine, Seoul, Korea

[2]Department of Medicine, Graduate School, Chung-Ang University, Seoul, Korea

[3]Department of Otorhinolaryngology-Head and Neck Surgery, Chung-Ang University College of Medicine, Seoul, Korea

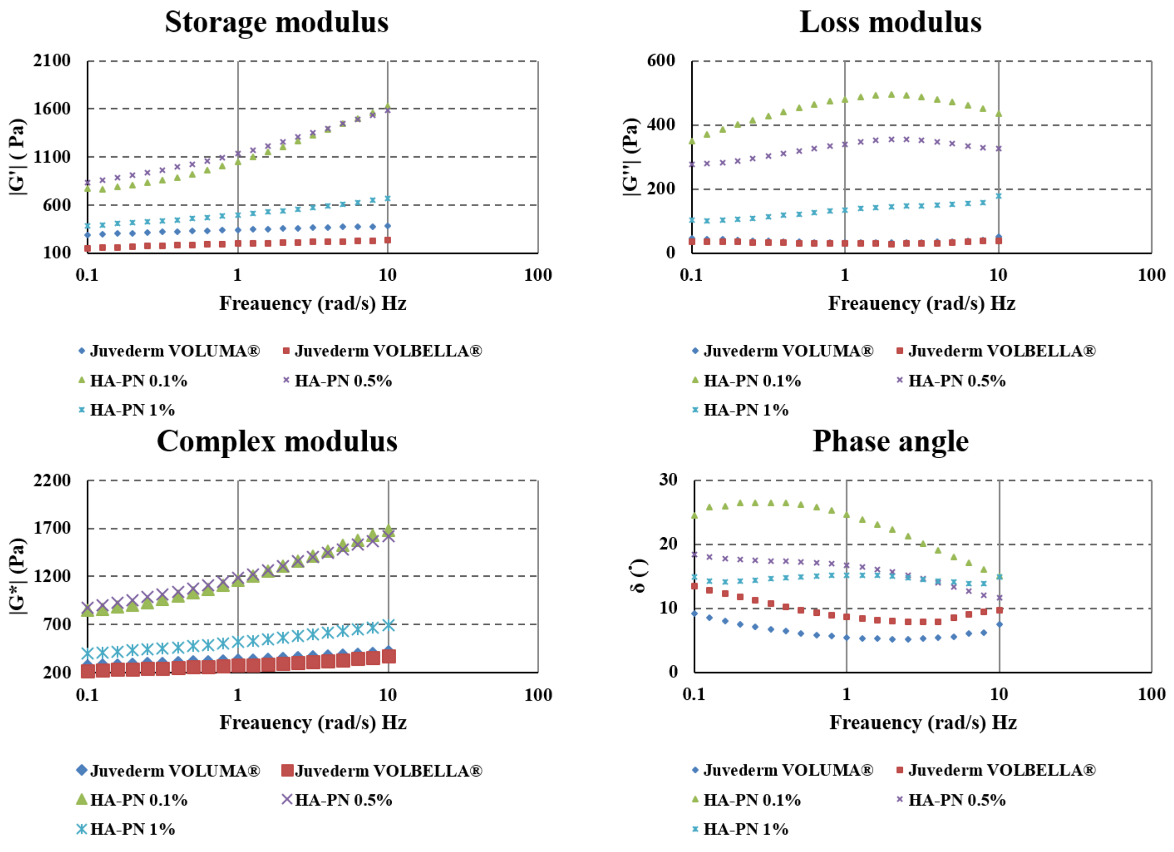

Supplementary Figure 1. Storage modulus (G′), loss modulus (G′′), complex modulus (G*), and phase angle (δ) values, obtained from the frequency sweep tests, as a function of frequency.
